# Proline Metabolism in WHO G4 Gliomas Is Altered as Compared to Unaffected Brain Tissue

**DOI:** 10.3390/cancers16020456

**Published:** 2024-01-21

**Authors:** Magdalena M. Sawicka, Karol Sawicki, Marek Jadeszko, Katarzyna Bielawska, Elżbieta Supruniuk, Joanna Reszeć, Izabela Prokop-Bielenia, Barbara Polityńska, Mateusz Jadeszko, Magdalena Rybaczek, Eryk Latoch, Krzysztof Gorbacz, Tomasz Łysoń, Wojciech Miltyk

**Affiliations:** 1 Department of Analysis and Bioanalysis of Medicines, Medical University of Bialystok, Mickiewicza 2D, 15-222 Bialystok, Poland; katarzyna.bielawska@umb.edu.pl (K.B.); wojciech.miltyk@umb.edu.pl (W.M.); 2 Department of Neurosurgery, Medical University of Bialystok, Skłodowskiej-Curie 24A, 15-276 Bialystok, Poland; karol.sawicki@umb.edu.pl (K.S.); mjadeszko63@gmail.com (M.J.); magdalenarybaczek@interia.pl (M.R.); krzysztofgorbacz@interia.pl (K.G.); tomasz.lyson@umb.edu.pl (T.Ł.); 3 Department of Physiology, Medical University of Bialystok, Mickiewicza 2C, 15-222 Bialystok, Poland; elzbieta.supruniuk@umb.edu.pl; 4 Department of Medical Pathomorphology, Medical University of Bialystok, Waszyngtona 13, 15-269 Bialystok, Poland; joannareszec@gmail.com; 5 Department of Medicinal Chemistry, Medical University of Bialystok, Mickiewicza 2D, 15-222 Bialystok, Poland; izabela.prokop-bielenia@umb.edu.pl; 6 Department of Psychology and Philosophy, Medical University of Bialystok, Szpitalna 37, 15-295 Bialystok, Poland; barbara.politynska-lewko@umb.edu.pl; 7 Department of Vascular Surgery and Transplantation, Medical University of Bialystok, Skłodowskiej-Curie 24A, 15-276 Bialystok, Poland; mzjo96@gmail.com; 8 Department of Pediatric Oncology and Hematology, Medical University of Bialystok, Waszyngtona 17, 15-274 Bialystok, Poland; eryk.latoch@umb.edu.pl

**Keywords:** glioblastoma, glioma, proline metabolism, proline oxidase, proline dehydrogenase, Δ^1^-pyrroline-5-carboxylate reductases, prolidase

## Abstract

**Simple Summary:**

Proline metabolism has been found to play an important role in neoplasms, but little is known about proline in gliomas or in the normal brain. This work investigates how the metabolism of proline in the brain and in gliomas of WHO grade 4 (GG4) may differ. A total of 20 pairs of samples were studied, consisting of both tumor and unaffected brain tissue, partially removed to make a surgical corridor. The levels of proline oxidase/proline dehydrogenase (POX/PRODH), Δ^1^-pyrroline-5-carboxylate reductases (PYCR1/2/3), prolidase (PEPD), and metalloproteinase-2 and -9 (MMP-2 and MMP-9) were measured. Proline concentration was evaluated. GG4 levels of POX/PRODH were found to be lower, while PYCR1, PEPD, and MMPs were significantly higher than in brain tissue. In GG4, proline concentration was 358% higher. The results confirm changes in proline metabolism in GG4, with a low-POX/PRODH/high-PYCR pattern like that in other neoplasms. High levels of PEPD and MMPs are in keeping with GG4 aggressiveness.

**Abstract:**

Proline metabolism has been identified as a significant player in several neoplasms, but knowledge of its role in gliomas is limited despite it providing a promising line of pursuit. Data on proline metabolism in the brain are somewhat historical. This study aims to investigate alterations of proline metabolism in gliomas of WHO grade 4 (GG4) in the context of the brain. A total of 20 pairs of samples were studied, consisting of excised tumor and unaffected brain tissue, obtained when partial brain resection was required to reach deep-seated lesions. Levels of proline oxidase/proline dehydrogenase (POX/PRODH), Δ^1^-pyrroline-5-carboxylate reductases (PYCR1/2/3), prolidase (PEPD), and metalloproteinases (MMP-2, MMP-9) were assessed, along with the concentration of proline and proline-related metabolites. In comparison to normal brain tissue, POX/PRODH expression in GG4 was found to be suppressed, while PYCR1 expression and activity of PEPD, MMP-2, and -9 were upregulated. The GG4 proline concentration was 358% higher. Hence, rewiring of the proline metabolism in GG4 was confirmed for the first time, with a low-POX/PRODH/high-PYCR profile. High PEPD and MMPs activity is in keeping with GG4-increased collagen turnover and local aggressiveness. Further studies on the mechanisms of the interplay between altered proline metabolism and the GG4 microenvironment are warranted.

## 1. Introduction

### 1.1. The Unsolved Problem of Glial Tumors

WHO grade 4 gliomas (GG4), i.e., glioblastoma IDH-wildtype WHO grade 4 and astrocytoma IDH-mutant WHO grade 4, is still incurable [1]. Despite the tremendous efforts of numerous research teams worldwide, little progress has been made with regard to therapy since the introduction of the Stupp regimen in 2005, consisting of a supra-maximal surgical resection with subsequent radiotherapy and concomitant chemotherapy based on temozolomide [2]. Despite the invasive nature of the surgical treatment, which is aimed at complete resection of the tumor, prognosis remains disappointingly pessimistic; for instance, to be diagnosed with glioblastoma (GBM), the most common primary brain tumor in the adult population, means to have merely 2 years of estimated survival [3]. Hope for new therapies is placed on understanding how the glioma metabolism deviates from that of a regular cell, aided with rapidly evolving experimental technologies addressing a whole spectrum of alterations in the metabolomic domain [4]. The importance of this direction is reflected in a new WHO classification of central nervous system (CNS) neoplasms, where glial tumors are differentiated according to the (unprecedented) hitherto unacknowledged contribution of molecular and genetic alterations [5].

### 1.2. Proline in CellularMetabolism

Studies over the past 40 years, initiated by Phang et al., have identified proline as being much more important than just a “twisted” building block that is necessary only as a helix breaker for shaping protein spatial structure [6]. Conversely, proline has been proven to play a key role in a vast array of processes, ranging from providing energy through anaplerosis and redox homeostasis to signaling, cell survival, and apoptosis [7]. This distinctive position of proline stems from the peptide bond contained within the pyrrolidine ring, a feature that is different from all other amino acids, making proline resistant to standard peptide-processing enzymes. Therefore, proline has evolved its own set of specific enzymes that, through a network of multi-layered connections, have gained a special position in cellular metabolism. Processing proline and its direct metabolite, Δ^1^-pyrroline-5-carboxylate (P5C), is performed solely by proline oxidase/proline dehydrogenase (POX/PRODH) and Δ^1^-pyrroline-5-carboxylate reductases (PYCRs). The exclusive role of POX/PRODH and PYCRs has led to the coining of the term “proline cycle”, referring to the interconversion between proline and P5C [8,9]. By means of P5C and its tautomer, glutamic-γ-semialdehyde, the proline cycle is placed at the strategic crossing of several metabolic pathways, e.g., the TCA cycle, urea cycle, pentose phosphate pathway, and is closely linked with glutamate and ornithine metabolism [10]. A distinctive feature of the proline cycle is the so-called redox shuttle, which, thanks to the subcellular location of proline cycle enzymes, uses reduction of P5C to proline through PYCRs (PYCR 1/2/3) for replenishing NADH or NADPH, and as a result, maintains redox homeostasis in conditions of oxidative stress [11].

Prolidase (PEPD) is another unique enzyme involved in proline metabolism which is vital for the functioning of the extracellular matrix (ECM). As opposed to the classic picture of ECM as an inert scaffold, there is growing evidence that the interplay between the ECM and cells plays a key regulatory role [12]. Hence, the fundamental ECM component, collagen, has also become recognized as a cellular metabolism regulator, responsible for cell migration, regeneration, and, specifically in the CNS, maintenance of synaptic connections [13]. In this context, an emerging role for PEPD is recognized as the only enzyme capable of cleaving C-terminus proline from dipeptides, derived mainly from the catabolism of collagen. Given the composition of collagen, up to 25% of which is made up of proline and hydroxyproline, PEPD is essential for collagen turnover [14].

### 1.3. Proline Pivots Rewired Tumor Metabolism

The position of proline-processing enzymes at the fulcrum of cellular metabolism has made them a convenient leverage point that has been exploited by multiple types of cancer. PYCRs are consistently upregulated in breast, lung, prostate, bladder, and liver cancers, and the correlation with poor prognosis in these conditions has been associated with proline cycle involvement in maintaining redox homeostasis [15]. Although in several cancers, POX is silenced, which has led to the hypothesis that POX is predominantly pro-apoptotic and that tumors with low POX activity are more likely to thrive, the body of evidence is not entirely unequivocal. Therefore, POX/PRODH is believed to play a dual, context-dependent role, being either pro-cancer or pro-apoptotic [16,17]. In the extracellular domain, PEPD and “upstream” metalloproteinases (MMP-2 and -9) have been shown to influence tumor metabolism and to shape microenvironment–tumor interplay. PEPD involvement in tumor growth, invasiveness, and spreading has been demonstrated. Apart from enzymatic function, PEPD is also a natural ligand of epidermal growth factor receptor (EGFR) and epidermal growth factor receptor 2 (HER2) and regulates the interferon α/β receptor. Moreover, PEPD controls the activity of p53, a potent tumor-suppressing protein, by providing storage for approximately 50% of this protein, which is released under stress conditions [18].

Although promising, the available body of evidence lacks robust experimental proof for alterations in proline metabolism in GG4; most of the studies were performed as in-vitro models and have not been verified in a clinical setting [19]. The most likely reason for this gap in knowledge is inherent to all studies on the CNS; for ethical reasons, it is very difficult to obtain samples of healthy brain tissue. Therefore, any comparison of proline metabolism in CNS tumors to unaffected brain tissue would be hampered by the absence of modern data concerning proline metabolism in the brain that could serve as a reliable reference point.

The aim of this work is to assess the basic constituents of proline metabolism in GG4 in comparison to unaffected brain tissue. To do so, we evaluated samples received from 20 patients undergoing surgical resection of GG4, in whom partial resection of the unaffected brain was a mandatory step to reaching a deep-seated tumor. Therefore, pairs of samples of both tumor and unaffected brain tissue were available to the study. Expression of proline cycle enzymes, i.e., POX/PRODH and PYCRs, were measured and compared with data mined from the Cancer Genome Atlas (TCGA). Additionally, we assessed the expression and activity of PEPD along with metalloproteinases of lesser specificity, i.e., MMP-2 and MMP-9. The concentration of proline together with selected amino acids and organic acids was measured. To the best of the authors’ knowledge, this is the first study evaluating aspects of proline metabolism both in tumor and unaffected brain samples, which, in addition, made it possible to compare tissue samples from the same patient in the series of cases examined.

## 2. Materials and Methods

### 2.1. Study Design

The study protocol was designed in accordance with the Declaration of Helsinki and obtained the approval of the Bioethics Committee of the Medical University of Bialystok (APK.002.461.2020). Participation in the study had no influence on therapy whatsoever. Whereas tumor removal is the first step in the standard treatment of GG4, brain specimens were harvested only if partial brain resection was an unavoidable step in creating a microsurgical corridor, required to reach a deep-seated lesion. All patients provided written informed consent for participation in the study prior to the collection of tissue samples and received information about the possibility of unconditioned consent withdrawal at any time. After obtaining the samples and histological diagnosis, participants’ identities were anonymized.

### 2.2. Material

**Figure 1:**
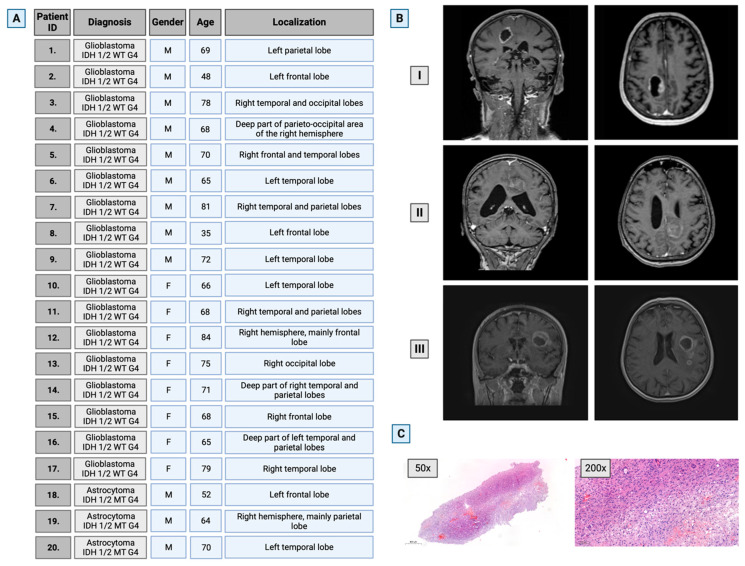
Characteristics of the selected study group. (**A**) Patients’ general information. (**B**) Representative brain images of three patients with GG4 (T1-weighted, contrast-enhanced 3T MRI scans in axial and coronal planes). (**C**) Representative hematoxylin–eosin staining for glioma WHO grade 4 at magnifications of 50× and 200×. M—male; F—female. Created with BioRender.com (accessed on 10 November 2023).

From a database consisting of material obtained from 147 patients who had undergone surgery in the Department of Neurosurgery, Medical University of Bialystok, Poland, during the years 2020–2022, 20 patients were identified, 12 male (median age 64 years) and 8 female (median age 72 years), from whom samples of both tumor and brain tissue had been collected. Only histologically proven cases of glioblastoma IDH-wild type WHO G4 and astrocytoma IDH-mutant WHO G4 that unambiguously met the WHO classification criteria for CNS tumors were included in the study. Details on patients’ demographics, tumor localization, and histopathological diagnosis are presented in Figure 1.

Diagnosis was made on the basis of signs, symptoms, and brain imaging, i.e., contrast-enhanced MRI. The decision to use surgical treatment, as well as the selected operating technique, approach, and instruments, had been at the discretion of the neurosurgery specialist and discussed with the patient prior to the surgery, along with all the information necessary for the patient to formulate informed consent to the surgical procedure. Only samples from treatment-naïve patients were included, i.e., patients who had received no treatment prior to surgery, with the exception of steroids for counteracting brain edema and anti-epileptic treatment if the patient so required. Therefore, patients who had undergone surgery for recurrent GG4 were excluded.

The goal of the surgery was safe and supra-maximal tumor excision, i.e., removing as much of the tumor mass as safely possible, preferably with a margin when applicable. All surgeries were performed by a neurosurgery specialist, with the patient under general anesthesia. Standard antibiotic and antithrombotic prophylaxis were applied. Positioning on the operating table was dictated by tumor location and the planned approach, with the patient’s head fixed in a Mayfield three-pin headholder. The exact tumor location and selected surgical corridor were confirmed with the aid of a navigation system that allowed the size of the craniotomy to be tailored appropriately.

Prior to tumor resection, a careful, microsurgical arachnoid and subpial dissection was performed within the limited area selected for corticotomy to secure samples of the unaffected brain. To provide samples of metabolically active tumors, during tumor resection, attention was paid to identifying areas of necrosis that were expected not to be suitable for examination in the study. While harvesting samples, care was taken to avoid unnecessary tissue damage, ensuring that use of bipolar coagulation was limited as far as possible. Immediately after surgical removal, samples were placed in sterile probes and deep-frozen at a temperature of −80 °C.

### 2.3. Real-Time PCR (RT-PCR)

Total RNA was extracted using the RNeasy Mini Kit (Qiagen, Hilden, Germany) according to the manufacturer’s protocol. RNA quantity and quality measurements were performed using spectrophotometry (at an absorbance OD ratio of 260/280 and 260/230). Total RNA (1 µg) served as a template for first-strand cDNA synthesis using the EvoScript universal cDNA master kit (Roche Molecular Systems, Boston, MA, USA). The following TaqMan probes were used: Hs04013270_cn for PEPD; Hs00132858_cn for POX/PRODH; Hs03050689_cn for PYCR2; Hs03692880_cn for PYCR3/PYCRL; Hs05414100_cn for MMP2; Hs00142484_cn for MMP9; Hs01060665_g1 for β-actin; Hs008943322_cn for GAPDH, Applied Biosystems, Waltham, MA, USA; Thermo Fisher Scientific, Inc., Waltham, MA, USA; qHsaCEP0054825 for PYCR1, BioRad, Hercules, CA, USA. TaqMan™ Gene Expression Master Mix (Applied Biosystems; Thermo Fisher Scientific, Inc.) was used to perform quantitative real-time polymerase chain reaction (qRT-PCR). The following thermocycling conditions were used: 50 °C for 2 min; 95 °C for 10 min; 40 cycles of 95 °C for 15 s; and 60 °C for 60 s. Each sample was analyzed in duplicate, and the expression of the above-mentioned genes was normalized to two housekeeping genes’ expression (GAPDH and β-actin) and calculated using the relative quantification method modified by Pfaffl [20]. Given the semi-quantitative nature of the assay, in each pair of samples, the one with brain tissue was considered as the control (set at 1), and the results for the tumor tissue sample were presented as a percentage of the control.

### 2.4. Mining The Cancer Genome Atlas (TCGA) and Genotype–Tissue Expression (GTEx) Databases for Gene Expression

To compare our transcriptomic data with those derived from a large patient cohort, we used publicly available TCGA RNA-sequencing data deposited in the Gene Expression Profiling Interactive Analysis (GEPIA) database. Validated gene expression values in GEPIA also combine the reference transcript levels from the Genotype–Tissue Expression project (GTEx) based on normal brain tissues [21].

### 2.5. Expression of Selected Proteins by Western Immunoblot (WB)

Tissue samples were homogenized in the presence of liquid nitrogen and subsequently suspended in RIPA buffer. Next, the samples were centrifuged for 30 min at 4 °C and 10,000 rpm. After centrifugation, the supernatant was transferred to another tube, avoiding the cell debris. Total protein content was determined using the BCA Protein Assay Kit according to the manufacturer’s instructions. The samples were stored at −80 °C until used. Equal amounts of each sample containing 25 µg of protein were denatured in the presence of Laemlli buffer (95 °C, 10 min) and separated by electrophoresis on SDS-PAGE gel (4–10%). Next, by use of semi-dry transfer (15 V, 30 min), the proteins were transported onto a nitrocellulose membrane followed by 1 h incubation with 5% non-fat dried milk (Santa Cruz Biotechnology, Dallas, TX, USA) in TBS-T (20 mM Tris, 150 mM NaCl, 0.1% Tween-20, pH 7.6). The membranes were then washed three times with TBS-T. Subsequently, the following primary antibodies were added at a dilution 1:1000 and held overnight at 4 °C on a rotator: anti-POX/PRODH antibody (St John’s Laboratory, London, UK); anti-PYCR1 and anti-GAPDH antibodies (Cell Signaling Technology, Danvers, MA, USA); anti-PYCR2, anti-PYCR3, and anti-PEPD antibodies (Biorbyt Ltd., Cambridge, UK). The membranes were then washed again (three times, 5 min) with TBS-T and incubated with secondary alkaline phosphatase-conjugated anti-rabbit antibody (Cell Signaling Technology) diluted 1:10,000 (2 h, RT). The visualization of the bands was gained by 1-StepTM NBT/BCIP Substrate Solution (Thermo Fisher Scientific, Waltham, MA, USA). Each band’s intensity was semi-quantitatively calculated with ImageJ ver. 1.53t software (https://imagej.nih.gov/ij/ (accessed on 10 November 2023), National Institutes of Health, Bethesda, MD, USA). The results for the proteins examined in each pair of samples are presented as a percentage of the control, set as 100%.

### 2.6. Immunohistochemistry (IHC)

The samples were fixed in formalin and embedded in paraffin for immunohistochemical evaluation. Immunohistochemical staining for expression of the examined proteins was performed on 4 μm sections cut from formalin-fixed paraffin-embedded tissues. Sections were deparaffinized in xylene and hydrated in a series of graded ethanol. Heat-induced antigen retrieval was performed with citrate buffer pH = 9.0 for RAGE and pH = 6.0. The characteristics of primary antibodies were the following: anti-PRODH antibody (ab203875); anti-PYCR1 antibody (ab279385); anti-PRD antibody (ab111851); anti-MMP2 antibody (ab86607); and anti-MMP9 antibody (ab76003) (Abcam, Cambridge, UK). The dilution was 1:150, respectively. The staining for examined proteins was performed using the EnVision visualization system (Dako/Agilent) linked with horseradish peroxidase. Diaminobenzidine (DAB) was used as the chromogen in all cases; the sections were then counterstained with hematoxylin. The intensity of immunostaining was evaluated in a random 10 fields under 50× and 200× magnification. The results were expressed as the percentage of cells with strong positive staining in both control brain tissue and within the tumor.

### 2.7. Gelatin Zymography Assay

The activity of MMP-2 and MMP-9 was assessed via the gelatin zymography assay as described by Wechselberger et al. [22]. Protein concentration was determined by use of the BCA Protein Assay Kit according to the manufacturer’s protocol. A total of 25 μg of protein per well was applied to gelatin 10% SDS-PAGE gels (1 mg/mL gelatin), and the electrophoresis was performed (120 V, 1 h 20 min). After that, the gels were washed three times with gelatinase renaturation buffer and then incubated in gelatinase reaction buffer at 37 °C (24 h). Coomassie Brilliant Blue dye was used for staining. Finally, the gels with visualized bands were scanned, and the intensity of bands was semi-quantitatively calculated with ImageJ ver. 1.53t software (https://imagej.nih.gov/ij/ (accessed on 10 November 2023), National Institutes of Health, Bethesda, MD, USA). The results for the proteins examined in each pair of samples are presented as a percentage of the control, set as 100%.

### 2.8. Determination of PEPD Activity

The activity of PEPD was determined according to the method described by Myara et al. based on the measurement of proline release from synthetic substrate, glycyl-proline [23]. Protein concentration was determined with the PierceTM BCA Protein Assay Kit (Thermo Fisher Scientific, Waltham, MA, USA) according to the manufacturer’s protocol. An equal volume of each sample (10 µL) was mixed with 50 mM Tris–HCl (pH 7.8) containing 2 mM MnCl_2_ and incubated for 24 h at 37 °C. Next, 94 mM glycyl-proline (substrate for PEPD) was applied and incubated for 1 h at 37 °C. The end of the process was achieved by 0.45 M ice-cold TCA. After centrifugation (10 min, 4000× *g*), the supernatant was transferred to fresh tubes and incubated with Chinard’s reagent (10 min, 90 °C) followed by incubation on ice for 15 min. Absorbance was read at 515 nm on the Genesys 10S UV-VIS Spectrophotometer (Thermo Fisher Scientific, Waltham, MA, USA). PEPD activity is presented as nanomoles of proline released from synthetic substrate, i.e., glycyl-proline, during 1 min and calculated per milligram of protein. The results were reported as a percentage of the control value.

### 2.9. Determination of Amino Acids and Organic Acids in Brain Homogenates

For LC-MS determination of amino acids (proline, glutamine, glutamic acid, ornithine, arginine) and organic acids (2-ketoglutarate, citrate, succinate, and oxaloacetate), about 15 mg of pulverized tissue was suspended in an ice-cold mixture of MeOH:MQW (4:1) spiked with internal standard (Pro-d_3_, C_IS_ = 30 µM, Supplementary Materials, Figure S1) to obtain 3% homogenates. Then samples were vortexed for 30 min at 4 °C, and after addition of ice-cold DCM (400 µL/15 mg of sample) and MQW (100 µL/15 mg of sample), the mixture was vortexed twice (for 1 min each time), while remaining in the ice-bath in between, and then incubated at −20 °C for 15 min and centrifuged at 15,000× *g* for 15 min at 4 °C. The 60 µL of supernatant was transferred to a tube, dried in a vacuum centrifuge concentrator, resuspended in 60 µL of MeOH, and centrifuged at 15,000× *g* for 15 min ([24] in our modification), and the supernatant was subjected to LC-MS analysis. The concentrations of metabolites were standardized to the total protein content.

LC-MS analyses were performed using a 1260 Infinity II HPLC coupled to a 6530 Q-TOF mass spectrometer with a dual ESI source (Agilent Technologies). Chromatographic separation of amino acids was conducted on a Poroshell 120 HILIC-Z column (2.1 × 100 mm, 2.7 µm particle size), Agilent) coupled to a Poroshell 120 HILIC (2.1 mm × 5 mm, 2.7 µm) guard column. The flow rate was 0.4 mL/min with an injection volume of 1 μL. The gradient elution of solvent A (10% 200 mM ammonium formate in MQW at pH = 3; 90% water) and solvent B (10% 200 mM ammonium formate in MQW at pH = 3; 90% acetonitrile) was programmed as follows: 0–10 min linear from 100% B to 70% B; 10–11 min from 70% B to 100% B; and 11–16 min with 100% B. Data were gathered in positive ionization mode (PI). The following dual ESI source parameters were applied: drying gas temperature of 300 °C; drying gas flow of 7 L/min; nebulizer pressure of 40 psig; fragmentor voltage of 100 V; and capillary voltage of 3000 V. The mass spectrometer operated with a mass range of 50–1000 *m*/*z* [25].

Chromatographic separation of organic acids was conducted on Synergi Fusion RP column (100 × 2.0 mm, 2.5 µm particle size, Phenomenex) coupled to Fusion-RB guard column (4 × 2.0 mm ID) at a flow rate of 0.25 mL/min at 30 °C. The gradient elution of solvent A (0.1% formic acid in water) and solvent B (0.1% formic acid in acetonitrile) was programmed as follows: 0–1.5 min with 98% solvent A; 1.5–4.0 min linear from 98% to 20% solvent A; 4.0–4.5 min with 20% solvent A; 4.5–5.0 min from 20% to 98% solvent A; and 5.0–12 min with 98% solvent A. Injection volume was constant and equal to 5 μL. Data were stored in negative ionization mode (NI). The following dual ESI source parameters were applied: drying gas temperature of 325 °C; drying gas flow of 12 L/min; nebulizer pressure of 45 psig; fragmentor voltage of 140 V; and capillary voltage of 3000 V. The mass spectrometer operated with a mass range of 50–1000 *m*/*z* ([26] in our modification). The mass spectrometer was calibrated and tuned according to the manufacturer’s instructions.

### 2.10. Statistical Analysis

The statistical analysis of the obtained results was performed using GraphPad Prism software version 8.0 (GraphPad Software, Inc., San Diego, CA, USA). The Shapiro–Wilk test for normality and the Levene test for homogeneity of variances were used to determine the application of parametric or non-parametric methods. Subsequently, the paired sample *t*-test or Wilcoxon test was used to compare the differences between the groups. Differences between TCGA and GTEx data were analyzed with a Mann–Whitney test. As a cutoff level for statistical significance, the *p* value of 0.05 was chosen.

## 3. Results

### 3.1. mRNA Expression of Genes Involved in the Proline Anabolic Axis and ECM Degradation Enzymes Is Enhanced

**Figure 2:**
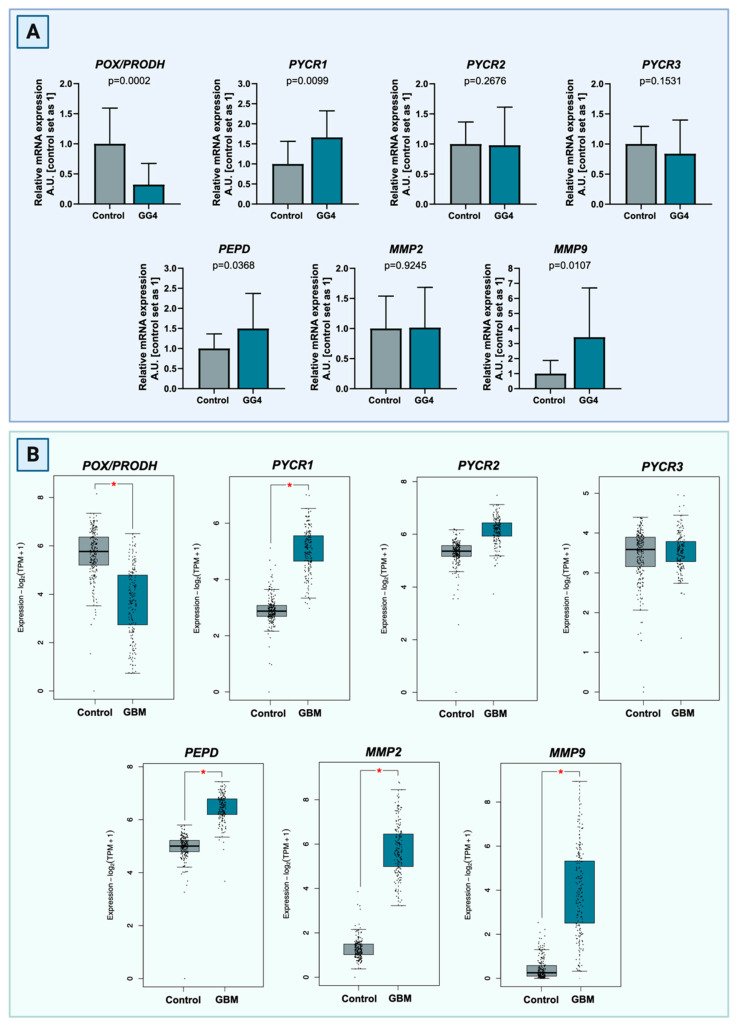
(**A**) Transcript levels of *POX/PRODH*, *PYCR1*, *PYCR2*, *PYCR3*, *PEPD*, *MMP-2*, and *MMP-9* compared to control samples of brain tissue. The results are presented in arbitrary units with the control set as 1. (**B**) Comparison of transcript levels of *POX/PRODH*, *PYCR1*, *PYCR2*, *PYCR3*, *PEPD*, *MMP-2*, and *MMP-9* genes between TCGA GBM cancer cohort (*n* = 163) and control brain tissue (*n* = 207) based on the GTEx samples. Differences are statistically significant at *p* < 0.05, marked with an asterisk (*). Created with BioRender.com (accessed on 10 November 2023).

In both brain and tumor samples, relative mRNA expression of key proline cycle enzymes, that is, *POX/PRODH*, *PYCR1*, *PYCR2*, and *PYCR3*, were evaluated, along with the expression of genes associated with ECM degradation, i.e., *PEPD*, *MMP-2*, and *MMP-9*. In comparison to the control brain tissue, expression of *POX/PRODH* mRNA was 68% lower in tumor tissue (*p* = 0.0002). Tumor *PYCR1* expression was 66% higher than in the control (*p* = 0.0099). Expression of *PYCR2* and *PYCR3* were relatively similar between groups and did not reach statistical significance (*p* = 0.2676 and *p* = 0.1531, respectively). mRNA expression of *PEPD* was significantly higher in GG4 samples (increase of 50%, *p* = 0.0368) as compared to the control. Expression of *MMP-9* in tumor tissue was significantly upregulated (increased of 242%, *p* = 0.0107) as opposed to the almost identical expression of MMP-2 (*p* = 0.9245;). In almost all cases, our results resembled the transcriptomic data deposited in TCGA and GTEx databases, which comprise the GBM cancer cohort (*n* = 163) and control brain tissue (*n* = 207). We noticed only one discrepancy, in *MMP2* expression level that was elevated in the TCGA cohort in comparison to our findings (Figure 2).

### 3.2. Expression of Proline Cycle Enzymes Favors Proline Biosynthesis Rather Than Utilization. Extracellular Matrix Catabolism Is Enhanced via Upregulation of Metalloproteinases and PEPD

To corroborate the results found in mRNA transcript, expression of respective proteins was verified with the Western immunoblot. Therefore, expression of POX/PRODH, PYCR1, PYCR2, PYCR3, and PEPD was evaluated in both tumor and brain. As in the RT-PCR assay, in each pair of samples, unaffected brain tissue is considered as the reference against which the result in tumor tissue is compared.

**Figure 3:**
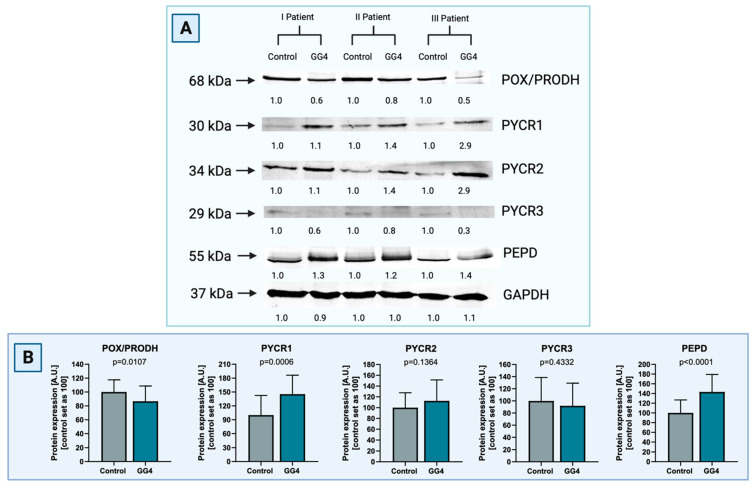
(**A**) Representative blot images of Western immunoblot for the enzymes of the proline cycle in GG4, i.e., POX/PRODH, PYCR1, PYCR2, PYCR3, and PEPD, compared to control samples of brain tissue (Supplementary Materials, Figure S2). Results are presented in arbitrary units with the control set as 1. GAPDH was used as a loading control. Densitometry of protein stains is presented under protein bands as intensity ratio versus control. (**B**) Relative GG4 expression of POX/PRODH, PYCR1, PYCR2, PYCR3, and PEPD compared to control samples of brain tissue. Results are presented in arbitrary units with the control set as 100. Created with BioRender.com (accessed on 10 November 2023).

The expression of POX/PRODH in GG4 was found to be decreased by 12% (*p* = 0.0107). Tumor PYCR1 was increased by 54% (*p* = 0.0006). Differences for PYCR2 (+13%) and PYCR3 (−8%) did not reach statistical significance, with *p* = 0.1364 and *p* = 0.4332, respectively. Expression of PEPD was significantly higher in tumor tissue in comparison to control brain tissue (+43%, *p* < 0.0001, Figure 3).

### 3.3. Increased Activity of PEPD and Metalloproteinases Indicates Remodeling of Extracellular Matrix

**Figure 4:**
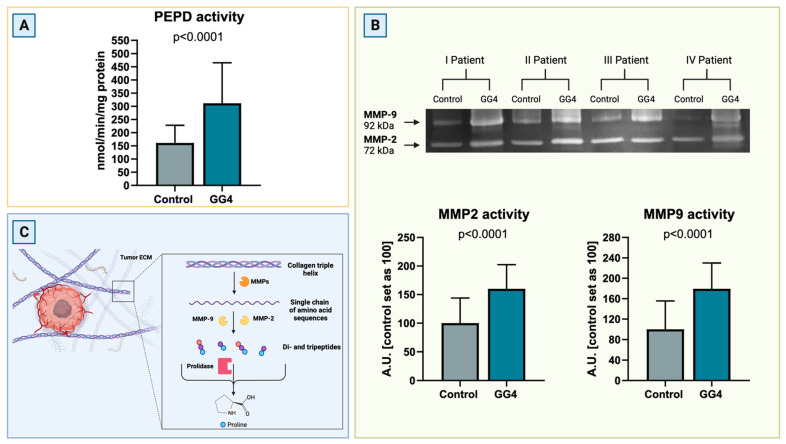
(**A**) PEPD activity in GG4 and brain, presented as an nmol of PEPD released within 1 min per milligram of protein. (**B**) Representative zymogram image (Supplementary Materials, Figure S3) of activity of MMP-2 and MMP-9 in the brain and GG4 and results of densitometry analysis for all samples. Results are presented in arbitrary units with the control set as 100. (**C**) Schematic route of collagen breakdown catalyzed by MMPs and PEPD. Created with BioRender.com (accessed on 10 November 2023).

To complete the results concerning potentially enhanced extracellular matrix degradation, the activity of PEPD and key MMPs, that is MMP-2 and MMP-9, was assessed. The activity of PEPD, the only enzyme capable of cleaving free proline from the C-terminus of dipeptides derived from collagen degradation, was nearly doubled in GG4 (+93%, *p* < 0.0001). The activities of MMP-2 and MMP-9 increased by 60% and 84%, respectively, and were much higher in tumor than in control brain tissue (*p* < 0.0001, Figure 4).

### 3.4. Enzymes Responsible for Proline Biosynthesis Are Highly Upregulated in GG4 Tissues, as Opposed to the Proline Catabolic Enzyme

**Figure 5:**
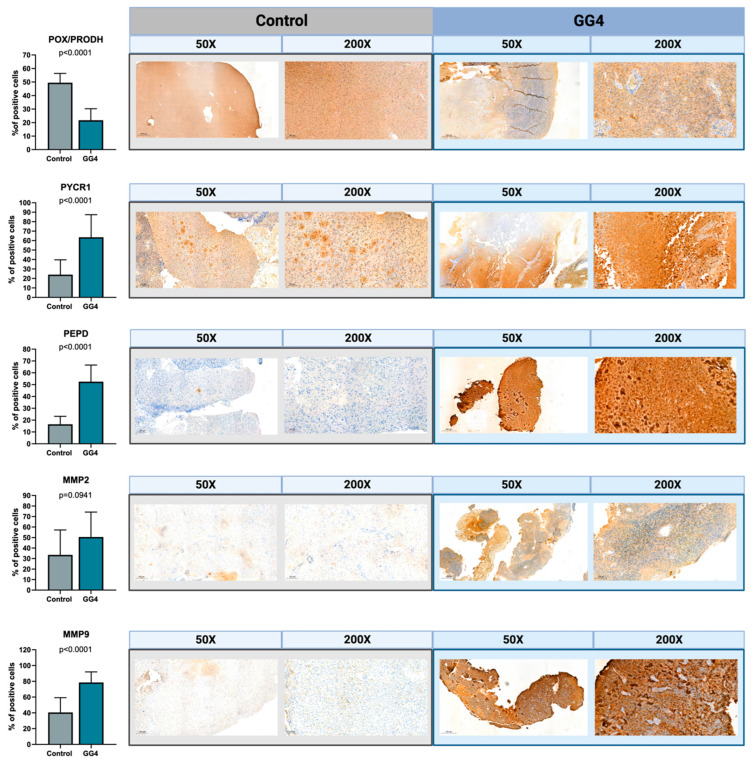
The expressions of POX/PRODH, PYCR1, PEPD, MMP-2, and MMP-9 in control brain tissue and GG4. The results are presented as the percentage of cells with strong positive staining. One selected image for both control and GG4 for each enzyme is presented at magnifications of 50× and 200×. Created with BioRender.com (accessed on 10 November 2023).

To confirm the results acquired from the techniques described above, we decided to perform immunohistochemical staining on formalin-fixed paraffin-embedded tissues. Additionally, staining served as a histopathology double-check, both for the tumor and control brain samples. Results corresponded to those obtained from RT-PCR and Western immunoblot. The percentage of cells with strong positive staining was significantly higher for tumor tissue when treated with PYCR1, PEPD, and MMP-9 antibodies (*p* < 0.0001) and lower when treated with POX/PRODH (*p* < 0.0001). The results for MMP-2 did not reach statistical significance (Figure 5).

### 3.5. Concentration of Proline and Proline-Related Metabolites

The mean proline concentration in brain samples was 10.1 ± 7.3 μM/g of protein (range from 1.0 to 23.7 μM/g of protein), whereas in all tumor samples, the level of proline was higher, reaching a mean concentration of 46.1 ± 34.95 μM/g of protein. In general, tumor proline concentration was higher by 358% compared to that found in brain tissue (*p* < 0.0001). The concentration of proline forerunners, glutamine and glutamate, was found to be downregulated. Glutamine concentration was slightly decreased (−12%, *p* = 0.7660), whereas glutamate concentration was markedly reduced (−63%, *p* < 0.0001). Metabolites engaged in the urea cycle, i.e., ornithine and arginine, had an increased concentration in tumor samples of 108% (*p* = 0.0016) and 39% (*p* = 0.0569), respectively. Also, the concentration of all studied TCA cycle metabolites was increased in tumor tissue, i.e., by 141% for 2-ketoglutarate (*p* = 0.0056), 56% for citrate (*p* = 0.0077), and 44% for oxaloacetate (*p* = 0.0038); a rise of 10% for succinate did not reach statistical significance (*p* = 0.1674, Figure 6).

## 4. Discussion

Two decades of studies have allowed us to identify the strategic position of proline in the reprogramming of tumor metabolism. Proline metabolism has been found to exert its influence through a network of links, ranging from anaplerotic detour of disrupted pathways and efficient aid in balancing redox homeostasis, to being a powerful modulator of both pro-survival and pro-apoptotic signals [12]. The presented study was designed to address the gap in knowledge concerning possible alterations of proline metabolism that might be present in astrocytic glial tumors with a high grade of malignancy. To overcome the paucity of modern data concerning proline in the healthy brain that might serve as a reference point, our study protocol included pairs of samples, i.e., brain and tumor, harvested from the same individual during one procedure. Our results show for the first time that the metabolism of proline in GG4 is significantly altered compared to control brain tissue. Here, the involvement of key proline-processing enzymes will be discussed, as well as future research perspectives.

In the studied brain–tumor pairs, POX/PRODH in GG4 is significantly suppressed, with unequivocal inter-modality agreement. Additionally, data on *POX/PRODH* gene expression mined from the TCGA corroborated our results. To the best of the authors’ knowledge, to date, there have not been any similar studies performed on both brain and tumor samples. Despite methodological differences, the results of the present study are in line with available data concerning POX/PRODH in GG4. A historical study by Loreck et al. on GBM cell cultures found POX/PRODH in GBM to be “low to absent” [27]. Similarly, a modern study by Shao et al. on cell cultures in an animal model also found POX/PRODH expression in GBM to be downregulated [28]. In the work of Panosyan et al. on a database of glioblastoma gene expression, POX/PRODH expression was found to be lower in GBM than in control samples [29].

Low POX/PRODH activity directs the proline cycle towards proline pooling and, as a result, may impair the restoration of glutamate and other linked metabolites of the TCA cycle or of the ornithine cycle. Although tumor ability to use proline for energy purposes may be impaired, Cappelletti et al. demonstrated that cell cultures of GBM with knocked-out POX/PRODH can adapt to compensate for the deficiency in glutamate, a key glial stimulant vital for GBM survival, in just 72 h [30]. Importantly, lower POX/PRODH activity may support tumor growth through a mechanism involving less-pronounced anti-tumor properties. The previously mentioned study of Shao et al. confirmed what has been known from legacy studies on colorectal cancer, namely, that POX/PRODH gene transcription is regulated by p53 and acts as a powerful tumor suppressor [28,31]. On the other hand, interesting results from Panosyan et al. have linked tumor aggressiveness with increased POX/PRODH activity; i.e., samples with high POX/PRODH transcription were associated with significantly shorter overall survival [29]. Indeed, studies on non-glial neoplasms have come to the conclusion that POX/PRODH might play an ambiguous, context-dependent role depending on factors that are yet to be established [16,32]. The best example for a dual POX/PRODH role is the influence exerted through ROS, a byproduct of POX/PRODH activity, that may be either pro-apoptotic or pro-survival, where the steering mechanism towards each path has not yet been finally established [17]. A number of pro-neoplastic POX/PRODH features have been demonstrated, e.g., POX/PRODH-driven supplementary energetic flux, stimulation of 5′AMP-activated protein kinase (AMPK) or peroxisome proliferator-activated receptors-γ (PPARγ), inhibition of T lymphocyte proliferation and function, or a kindling pro-inflammatory milieu [33]. By contrast, a study by Liu et al. demonstrated that POX/PRODH may exert a suppressive impact on the cyclooxygenase-2 (COX-2) enzyme, counteracting development of the tumor microenvironment, dependent on the stimulatory influence of prostaglandin [34]. Similarly, Tołoczko-Iwaniuk, studying oral squamous cell carcinoma, demonstrated that a COX-2 inhibitor, celecoxib, leads to apoptosis through increased levels of POX/PRODH [35]. Overall, the ongoing debate concerning the role of POX/PRODH in cancer has led to the tentative conclusion that for a tumor to thrive, POX/PRODH must be silenced, a position supported by studies such as that by Liu et al., where POX/PRODH was found to be downregulated in a large group of various cancers paired with unaffected parent tissue used as a reference [36].

Out of the three studied PYCR isoforms, only PYCR1 turned out to be uniformly upregulated in tumor tissue, which was corroborated by the three assays performed—RT-PCR, Western immunoblot and IHC; minor differences noted in PYCR2 and PYCR3 did not yield statistical significance. These results mirrored the pattern for *PYCRs* gene expression found in data extracted from the TCGA. To the authors’ knowledge, information pertaining to PYCRs in glial tumors, specifically PYCR1, may be found only in the study of Hollinshead et al., conducted in vitro on oligodendroglioma WHO G3 cells with and without isocitrate dehydrogenase (IDH) mutation. Cultures bearing the IDH mutation, which resulted in hampering the TCA cycle, were shown to bypass metabolic blockage by means of the so-called “proline shuttle”, i.e., increased proline synthesis by PYCR1 used as a means of oxidizing excessive NADH, therefore restoring the mitochondrial redox balance. As a control, oligodendroglioma cultures without the IDH mutation were used, in which PYCR1 activity was less pronounced [37]. Interestingly, to produce proline via PYCR1, glutamate-derived P5C is primarily used; therefore, cellular glutamate, known for its influence both on glia and glioma cells, is depleted [38,39].

In the study of Hollinshead et al., as well as in the present study, the relevance of PYCR1 in glial neoplasms is emphasized. However, the results of both studies cannot be directly compared to each other due to differences in study groups (oligodendroglioma WHO G3 vs. GG4) and the selection of different reference points, i.e., IDH wildtype tumor vs. unaffected brain tissue. Additionally, the study of Hollinshead et al. was designed specifically to seek the involvement of the proline cycle in re-establishing redox balance disturbed by IDH mutation. Although relation to IDH mutation is of utmost importance in understanding GG4 metabolic alteration, our present study does not provide sufficient data to allow clinical verification of the possible relationship between IDH and proline metabolism. Among the 20 pairs of samples presented here, IDH mutation was found in only 3 cases; for this reason, drawing conclusions about PYCR1 activity and different IDH mutation statuses is not possible. Given the importance of IDH status in astrocytic tumor prognosis, this aspect calls for further studies with a larger cohort.

Upregulation of PYCR1 has been widely documented in general oncology. A study by Bogner et al. examined data from the gene expression databases of 28 cancer types and found that PYCR1 is universally elevated. For this reason, PYCR1 was proposed as a new, promising target for future therapies. Data on PYCR2 and PYCR3 show that their expression varies in different types of tumors [40]. Similarly, no clear pattern can be confirmed from the results of the present study; whereas PYCR1 is strongly upregulated, differences in PYCR2 and PYCR3 are less pronounced and turned out not to be significant. It is worth noting that whereas PYCR1 and PYCR2 are located within mitochondria, PYCR3 is found only in cytosol. Therefore, PYCR3 utilizes a separate P5C pool, displays different substrate preferences—that is, ornithine and NADPH—and is involved in different metabolic pathways [7]. An important point has been noted by De Ingeniis et al. in a study performed on a model of melanoma cell lines. The authors demonstrated that even though the measured differences in expression of PYCR2 and PYCR3 in tumor tissues lacked statistical significance, the influence exerted on tumor metabolism was found to be relevant, mainly due to the cytosolic localization of PYCR3 and the production of NADPH, resulting in the activation of PPP [38]. Therefore, although our results indicate significant differences only in the PYCR1 isoform, it would seem appropriate to refrain from definite conclusions dismissing the role of PYCR2 and PYCR3 in gliomas until the wider context of the multi-layered metabolic network involved is better understood.

When compared to brain tissue, PEPD was found to be uniformly strongly upregulated in GG4, which has been confirmed in all the assays used in the study. To date, only two reports concerning PEPD in GG4 are available. Verma et al. demonstrate that PEPD activity in GBM is higher than in control samples of cadaver brain tissue, and PEPD serum activity in GBM patients is elevated [41]. Conversely, a study by Gönullu et al. performed only on serum samples from GBM patients reported results contradictory to those of Verma et al. [42]. Additionally, we confirmed the upregulation of proteinases placed “upstream” to PEPD, i.e., MMP-2 and MMP-9. Therefore, activation of an entire collagen breakdown axis in GG4 was confirmed; while MMP-2 and -9 begin the multi-step process of collagen degradation, PEPD completes fragmentation by cleaving free proline from the C-terminus of di- and tripeptides. Increased degradation of collagen might be interpreted not only as catabolic deconstruction, driven by the voracious energetic appetite of GG4; GG4 are also known to have higher amounts of collagen than the brain tissue itself [43]. Thus, increased collagen breakdown may represent enhanced collagen turnover, aimed at ECM remodeling. As a result, tumor-driven changes in ECM might be responsible for shaping the pro-tumoral milieu and, importantly, enabling GG4 invasiveness, manifesting itself through devastating brain infiltration abilities. Although information about PEPD is limited, in several studies, the role of MMP-2 and MMP-9 has been underlined, e.g., finding positive correlations between MMPs levels and increasing grades of glioma malignancy [44,45] associated with invasion, recurrences, poor prognosis [46,47,48], and shorter survival [49]. We hypothesize that the aforementioned findings concerning MMPs might turn out to be true for PEPD as well, given the fundamental role of PEPD as a last and obligatory step in collagen catabolism.

Apart from its enzymatic role, PEPD displays potent regulatory abilities associated with EGFR and HER2 receptors and the regulatory activity of p53 [18]. Unfortunately, the literature is lacking in reports verifying this type of PEPD activity in GG4, although the importance of both p53 and EGFR in GG4 have been confirmed [50,51,52].

In the samples examined, the concentration of proline in GG4 was found to be significantly elevated. Increases in proline levels seem to be the result of two independent processes, i.e., enhanced de novo synthesis and intensified ECM catabolism. Increased levels of PYCR1 in GG4 promote proline synthesis, mainly at the expense of cellular glutamate, as demonstrated by De Ingeniis et al. in a model of melanoma cells, where glutamate was shown to be the substrate of choice for PYCR1, with notably lower affinity for ornithine [38]. PYCR1 substrate preference is in line with the results of the present study, where in GG4, low concentrations of glutamate were present despite high levels of ornithine. Although glutamate is of well-documented importance to GG4, for instance, as a vital energy source available in hypoxic stress conditions and as an excitatory glial stimulant with several regulatory and signaling properties [53], in the literature on GBM glutamate concentration, evaluated primarily on the basis of magnetic resonance spectroscopy (MRS) and brain fluid microdialysis, different and ambiguous results have been reported [54]. We hypothesize that the trend observed in our results, i.e., PYCR1-driven glutamate-to-proline routing, might suggest that GG4 metabolism favors maintaining redox homeostasis over the pro-tumoral role of glutamate. From this point of view, proline might be perceived as an oncometabolite, which accumulates due to high PYCR1 and low POX/PRODH activity. This explanation is in keeping with the known appetite for glutamate exhibited by GG4 cells, which, for this reason, is dubbed a “glutamate-addicted tumor” since glutamate is the main fuel for PYCR1 to restitute NAD^+^ [39]. On the other hand, low levels of glutamate may result from using glutamate for other purposes as well, such as anaplerosis of the TCA cycle, whose metabolites, such as 2-KG, oxaloacetate, succinate, and citrate, were also found to be significantly upregulated in the samples studied. Overall, we believe that the reported decrease in glutamate concentration does not have to be inconsistent with what is known about the role of glutamate in signaling glioma; it might rather reflect imbalanced glutamate turnover in gliomas, favoring routing large quantities of glutamate to proline through PYCR1 in an attempt to maintain redox balance. We hypothesize that even with a decrease in the total concentration, in specific cellular compartments, the amount of glutamate might still be sufficient to maintain signaling on relatively normal or even increased levels.

Simultaneously, an increase in the activity of the ECM catabolic axis, i.e., PEPD and MMPs, provides a flux of proline from the catabolism of collagen. The resulting situation in GG4 may be similar to that modeled by Ferreira et al., in which GBM cultures were treated with increasing concentrations of proline. Interestingly, the authors found an overall pro-tumoral influence of proline with regard to GBM, with promotion of nuclear factor-κB (NF-κB) signaling and ROS production, while no increase in apoptosis or cytotoxicity was found [55]. Given the high concentration of proline in the samples presented in this paper, the experiment conducted by Ferreira et al. would seem to yield similar results to the in vivo conditions reported here. Thus, further studies aimed at verifying the mechanisms underlying the influence of proline in the oncogenesis of GG4 would appear to be well founded.

The results of the present study add to the promising evidence in the field of general oncology, suggesting that targeting enzymes of the proline cycle might be therapeutically beneficial. For instance, diminishing PYCR1 activity has been found to increase the cytotoxicity of doxorubicin against MCF-7 breast cancer cells [56]. In the case of lung adenocarcinoma cell cultures, PYCR1 silencing has been associated with increased cisplatin sensitivity [57]. Interesting results concerning POX/PRODH have come from an experiment on C32 melanoma cell cultures, where metformin stimulation led to decreased cell viability via an increase in POX/PRODH [58].

Hence, both PYCR1 silencing and POX/PRODH stimulation hold promise for future management strategies in GG4. Equally, knowledge concerning alterations in proline metabolism might serve to improve diagnostics. In GG4, where diagnosis is usually made at a late symptomatic stage of the disease and no robust biomarkers have been identified to date, research towards a non-invasive, easily-accessible diagnostic test, preferably based on studying body fluids, is of crucial importance.

## 5. Conclusions

**Figure 7:**
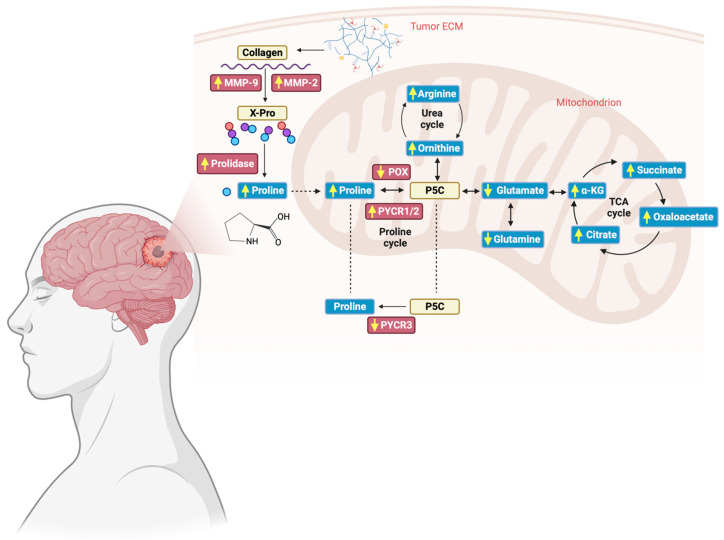
Schematic representation of alterations in the proline cycle and collagen breakdown found in GG4. Enzymes investigated in the study are presented in red boxes, whereas metabolites, i.e., amino acids and organic acids, are placed in blue boxes. Yellow arrows represent how the level of the examined substances has changed in comparison to unaffected brain tissue. Created with BioRender.com (accessed on 10 November 2023).

The results of this study provide strong support for the contention that proline metabolism is remodeled in GG4, involving increased proline synthesis by PYCR1, downregulation of POX/PRODH, and enhancement of PEPD and MMPs activity (Figure 7). We believe that the results presented are a robust starting point for future studies investigating how the metabolism of proline affects GG4 and whether that knowledge might be useful in the therapeutic struggle against glial tumors.

## Figures and Tables

**Figure 6:**
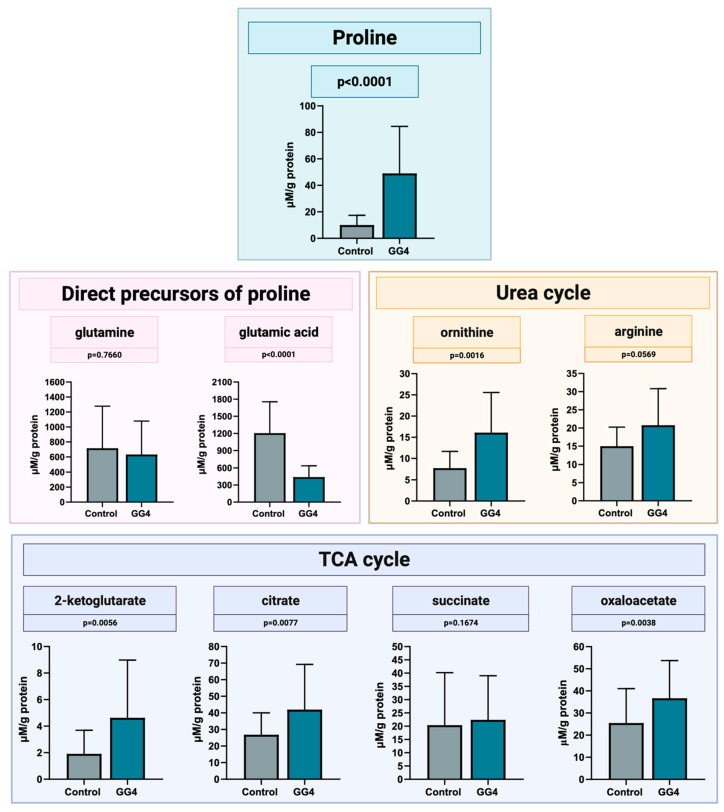
Concentration of proline, its direct precursors, and metabolites of urea and the TCA cycle, measured by LC-MS in GG4 and control brain samples. Created with BioRender.com (accessed on 10 November 2023).

## Data Availability

Data presented are contained within the article. The authors can be contacted for data; requests will only be considered for academic collaboration purposes.

## Supplementary Materials

The following supporting information can be downloaded at https://www.mdpi.com/article/10.3390/cancers16020456/s1, Figure S1: LC-MS chromatogram of proline d3 (Pro-d3, internal standard, Cis = 30 μM); Figure S2: The POX/PRODH (68 kDa), PYCR1 (30 kDa), PYCR2 (34 kDa), PYCR3 (29 kDa), PEPD (55 kDa) and GAPDH (37 kDa) expression of three representative patients; Figure S3: The MMP-2 and MMP-9 activity of four representative patients.
