# Supplementary material for: Proline Metabolism in WHO G4 Gliomas Is Altered as Compared to Unaffected Brain Tissue
Source: Cancers (Basel). 2024 Jan 21;16(2):456. doi: 10.3390/cancers16020456 (PMC10814259; doi:10.3390/cancers16020456)
Supplement: Supplementary file 1 [file cancers-16-00456-s001.zip › cancers-2788451-supplementary.pdf]

Supplementary Material Article

Proline Metabolism in WHO G4 Gliomas is Altered as Compared to Unaffected Brain Tissue.

Magdalena M. Sawicka[1*], Karol Sawicki[2], Marek Jadeszko[2], Katarzyna Bielawska[1], Elżbieta Supruniuk[3], Joanna Reszeć[4], Izabela Prokop-Bielenia[5], Barbara Polityńska[6], Mateusz Jadeszko[7], Magdalena Rybaczek[2], Eryk Latoch[8], Krzysztof Gorbacz[2], Tomasz Łysoń[2] and Wojciech Miltyk[1]

[1] Department of Analysis and Bioanalysis of Medicines, Medical University of Bialystok, Mickiewicza 2D, 15-222 Bialystok, Poland, magdalena.sawicka@umb.edu.pl (M.M.S.); katarzyna.bielawska@umb.edu.pl (K.B.); wojciech.miltyk@umb.edu.pl (W.M.)

[2] Department of Neurosurgery, Medical University of Bialystok, Skłodowskiej-Curie 24A, 15-276 Bialystok, Poland, karol.sawicki@umb.edu.pl (K.S.); mjadeszko63@gmail.com (M.J.[2]); magdalenarybaczek@interia.pl (M.R.); krzysztofgorbacz@interia.pl (K.G.); tomasz.lyson@umb.edu.pl (T.Ł.)

[3] Department of Physiology, Medical University of Bialystok, Mickiewicza 2C, 15-222 Bialystok, Poland, elzbieta.supruniuk@umb.edu.pl (E.S.)

[4] Department of Medical Pathomorphology, Medical University of Bialystok, Waszyngtona 13, 15-269 Bialystok, Poland, joannareszec@gmail.com (J.R.)

[5] Department of Medicinal Chemistry, Medical University of Bialystok, Mickiewicza 2D, 15-222 Bialystok, Poland, izabela.prokop-bielenia@umb.edu.pl (I.P-B.)

[6] Department of Psychology and Philosophy, Medical University of Bialystok, Szpitalna 37, 15-295 Bialystok, Poland, barbara.politynska-lewko@umb.edu.pl (B.P.)

[7] Department of Vascular Surgery and Transplantation, Medical University of Bialystok, Skłodowskiej-Curie 24A, 15-276 Bialystok, Poland, mzjo96@gmail.com (M.J.[7])

[8] Department of Pediatric Oncology and Hematology, Medical University of Bialystok, Waszyngtona 17, 15-274 Bialystok, Poland, eryk.latoch@umb.edu.pl (E.L.)

[*] Corresponding author.: magdalena.sawicka@umb.edu.pl

Description of data: Blots, zymograms and LC-MS chromatograms are described in the result section.

Supplementary data analysis presented: LC-MS chromatogram of internal standard (proline d3, **Figure 6.**), representative blots from Western Immunoblot (**Figure 3A)**, representative zymogram from zymography (**Figure 4B)**.

1. LC-MS chromatogram of internal standard (proline d_3).

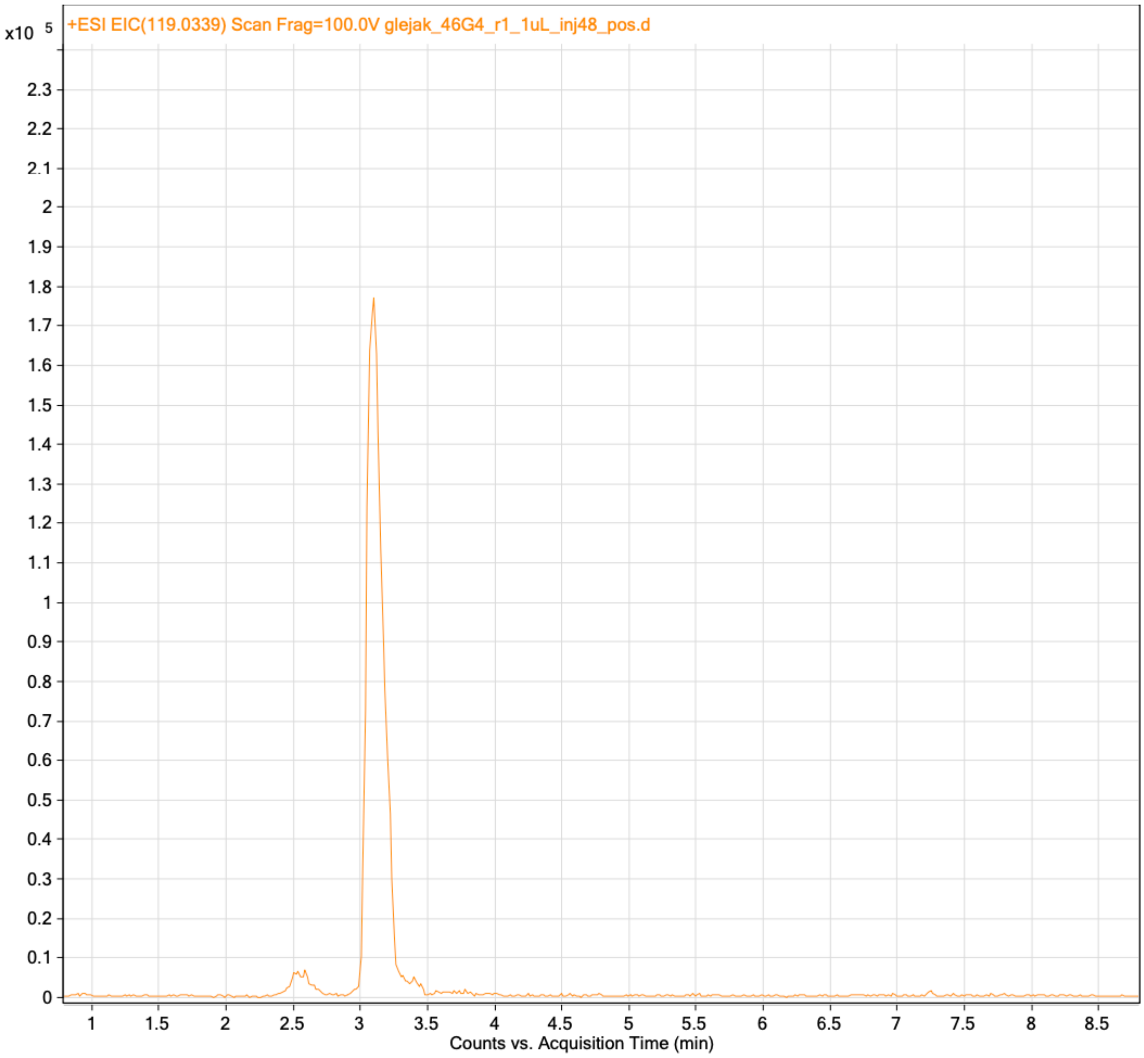

Figure S1. LC-MS chromatogram of proline d_3 (Pro-d_3, internal standard, C_{is}=30µM)

2. Representative blots from Western Immunoblot analysis presented in Figure 3A.

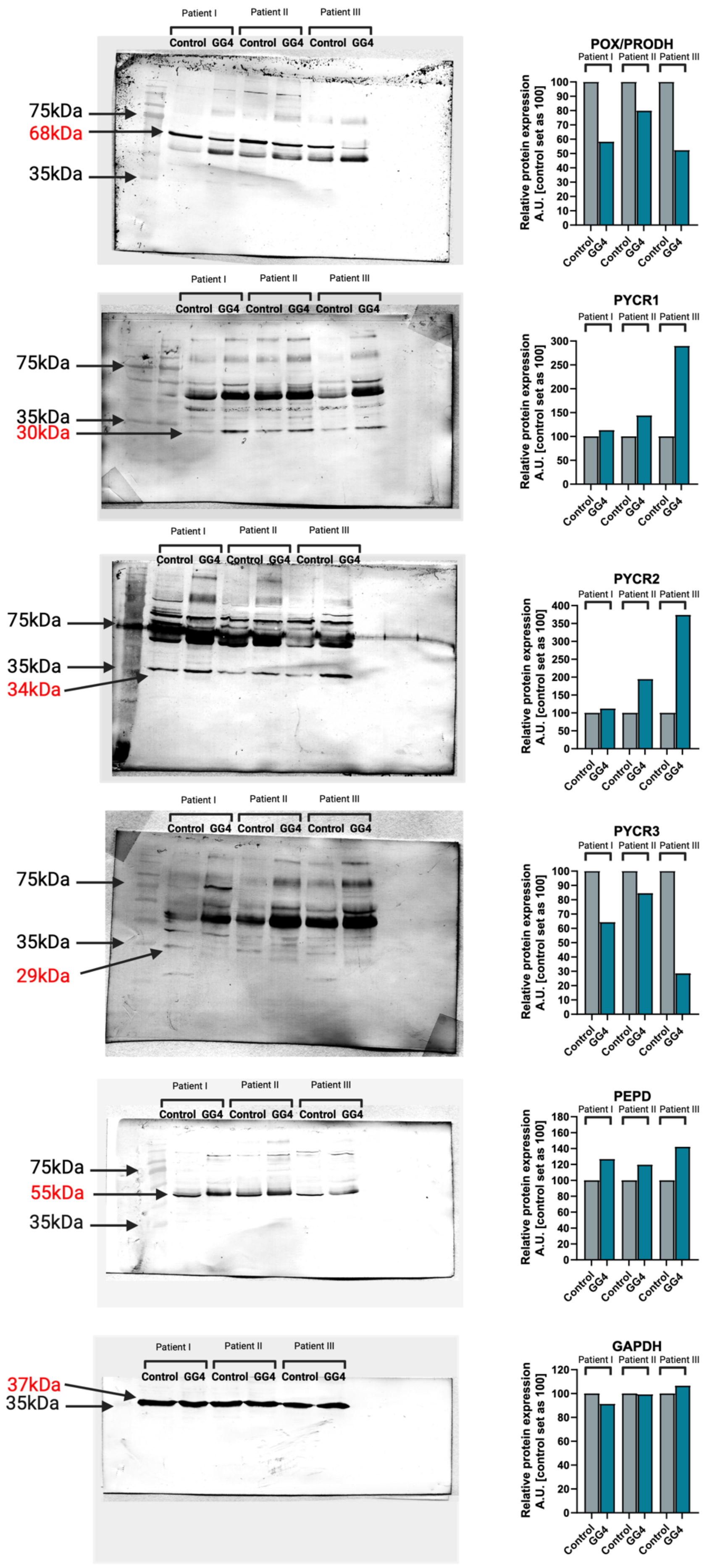

Figure S2. The POX/PRODH (68kDa), PYCR1 (30kDa), PYCR2 (34kDa), PYCR3 (29kDa), PEPD (55kDa) and GAPDH (37kDa) expression of three representative patients. GAPDH expression was used as a loading control. The WB bands intensity of representative blots was quantified by densitometry with ImageJ software (https://imagej.nih.gov/ij/, National Institutes of Health, Bethesda, MD, USA). Created with BioRender.com

3. **Representative zymogram from gelatin zymography analysis presented in Figure 4B**.

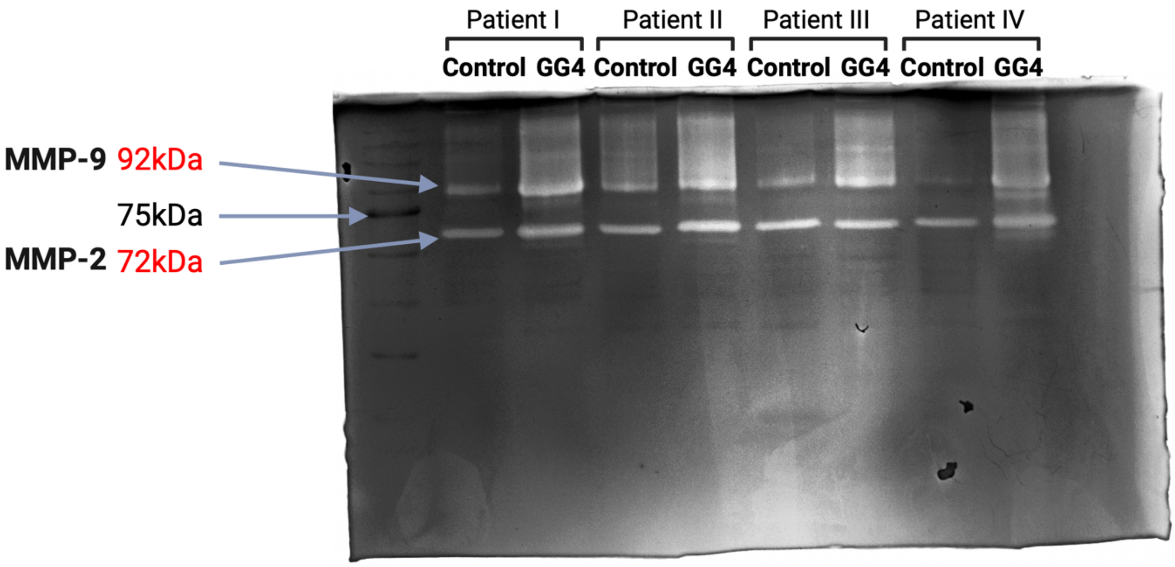

Figure S3. The MMP-2 and MMP-9 activity of four representative patients. The intensity of bands was semi-quantitatively calculated with ImageJ software (https://imagej.nih.gov/ij/, National Institutes of Health, Bethesda, MD, USA). Created with BioRender.com
